# Common CT Findings of Novel Coronavirus Disease 2019 (COVID-19): A Case Series

**DOI:** 10.7759/cureus.7434

**Published:** 2020-03-27

**Authors:** Pooya Torkian, Naghi Ramezani, Pejman Kiani, Michael R Bax, Shahram Akhlaghpoor

**Affiliations:** 1 Radiology, Shahid Beheshti University of Medical Sciences, Tehran, IRN; 2 Radiology, Pars Hospital, Rasht, IRN; 3 Neuroscience and Addiction Studies, School of Advanced Technologies in Medicine, Tehran University of Medical Sciences, Tehran, IRN; 4 Biomedical Engineering, California Institute of Computer Assisted Surgery, Los Altos, USA; 5 Radiology, Pardis Noor Medical Imaging Center, Tehran, IRN

**Keywords:** case series, chest ct, consolidation, covid-19, crazy paving, ground-glass opacity, novel coronavirus, radiology, respiratory disease

## Abstract

Given the highly infectious nature of the coronavirus disease 2019 (COVID-19) virus and the lack of proven specific therapeutic drugs and licensed vaccines effective against it, early diagnosis of the disease is of paramount importance. The common chest CT imaging of confirmed COVID-19 cases is discussed here, which shows ground-glass opacity, crazy paving, and consolidation.

## Introduction

In late December 2019 with the outbreak of coronavirus disease 2019 (COVID-19) and an exponentially growing death toll in Wuhan, China, a state of emergency was declared [1]. Iran, n subsequent epicenter of COVID-19, announced a total of 724 deaths due to COVID-19 as of March 16, 2020 [2]. Given the highly infectious nature of the virus and the lack of proven specific therapeutic drugs and licensed vaccines effective against COVID-19, early diagnosis of the disease is of paramount importance [3]. As confirmed COVID-19 cases are being diagnosed all over the world, radiologists are key to prompt clinical diagnosis and understanding of the common imaging manifestation of the disease as they may encounter suspected cases prior to other clinicians. The experience of affected countries shows that CT can play a pivotal role for pneumonia screening and diagnosis by producing fine specific details. The common chest CT imaging of confirmed COVID-19 cases is reported here to aid prompt clinical diagnosis.

## Case presentation

Case 1

Ground-glass Opacity

Ground-glass opacity (GGO) is frequently observed due to enhanced lung attenuation without obscuration of underlying vessels. In GGO, alveoli are partially filled with fluid which appears gray on CT images. The distribution of GGO lesions in COVID-19 tends to be peripheral, bilateral, and along the pleura and bronchovascular bundles [4-6].

**Figure 1:**
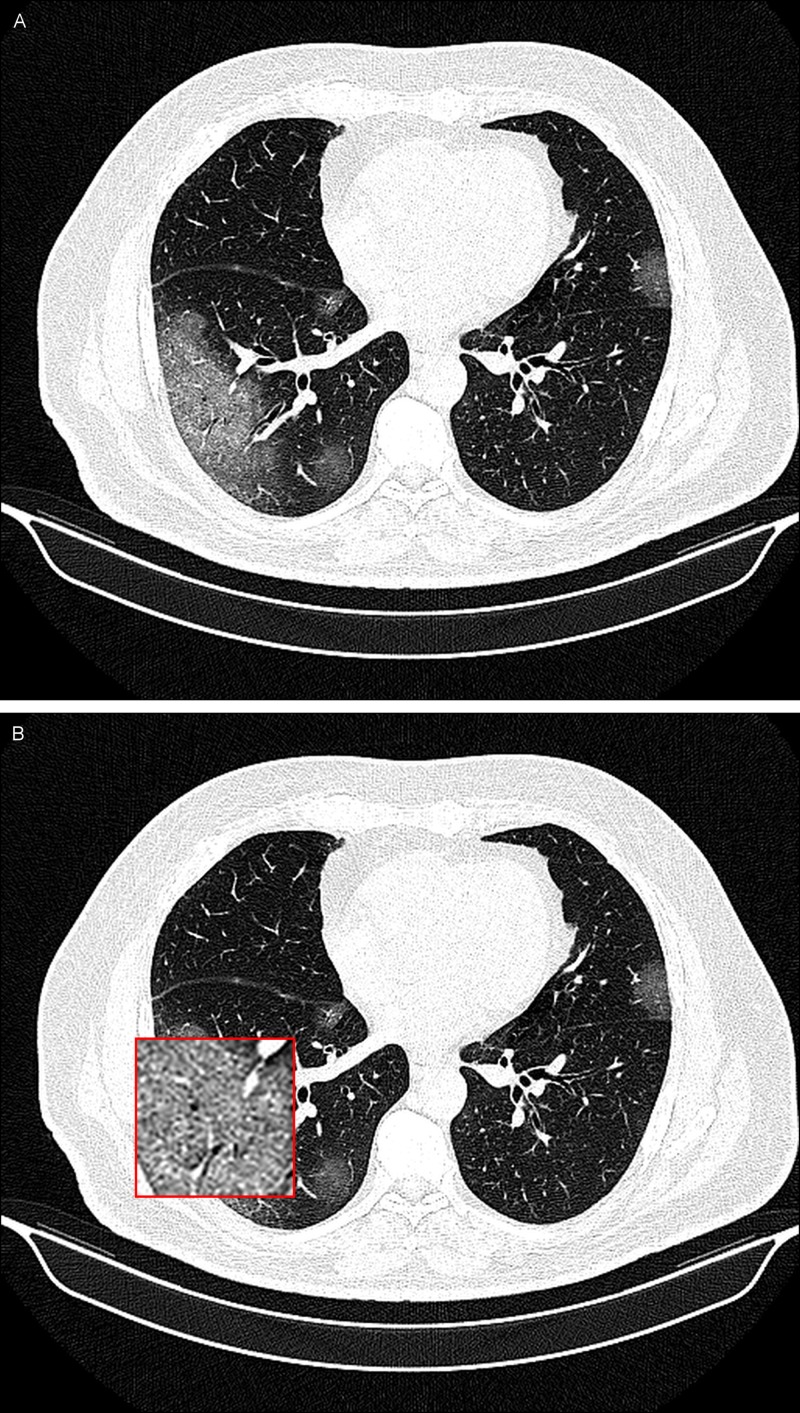
A 45-year-old woman with COVID-19 infection. A noncontrast-enhanced CT image of the lungs demonstrating bilateral patchy ill-defined ground-glass opacities (GGOs) in posterior segments of the lungs (A) and an inset magnified view of the lesion for better delineation at the location of GGO in the right lower lobe of the lung (B).

A 45-year-old woman from the Rasht provincial hospital presented three days after the onset of fever (38.2°C), cough, and chills. She did not reveal any history of sick contacts in the family or underlying lung disease. Unenhanced chest CT images at her presentation showed patchy ill-defined GGOs as an archetypal response to acute lung injury in the left and right lower lobes of the lung (Figure 1).

Case 2

Crazy Paving

Following lung involvement in COVID-19, the thickened interlobular septa and intralobular lines laid over a background of GGO known as crazy paving can be observed.

**Figure 2:**
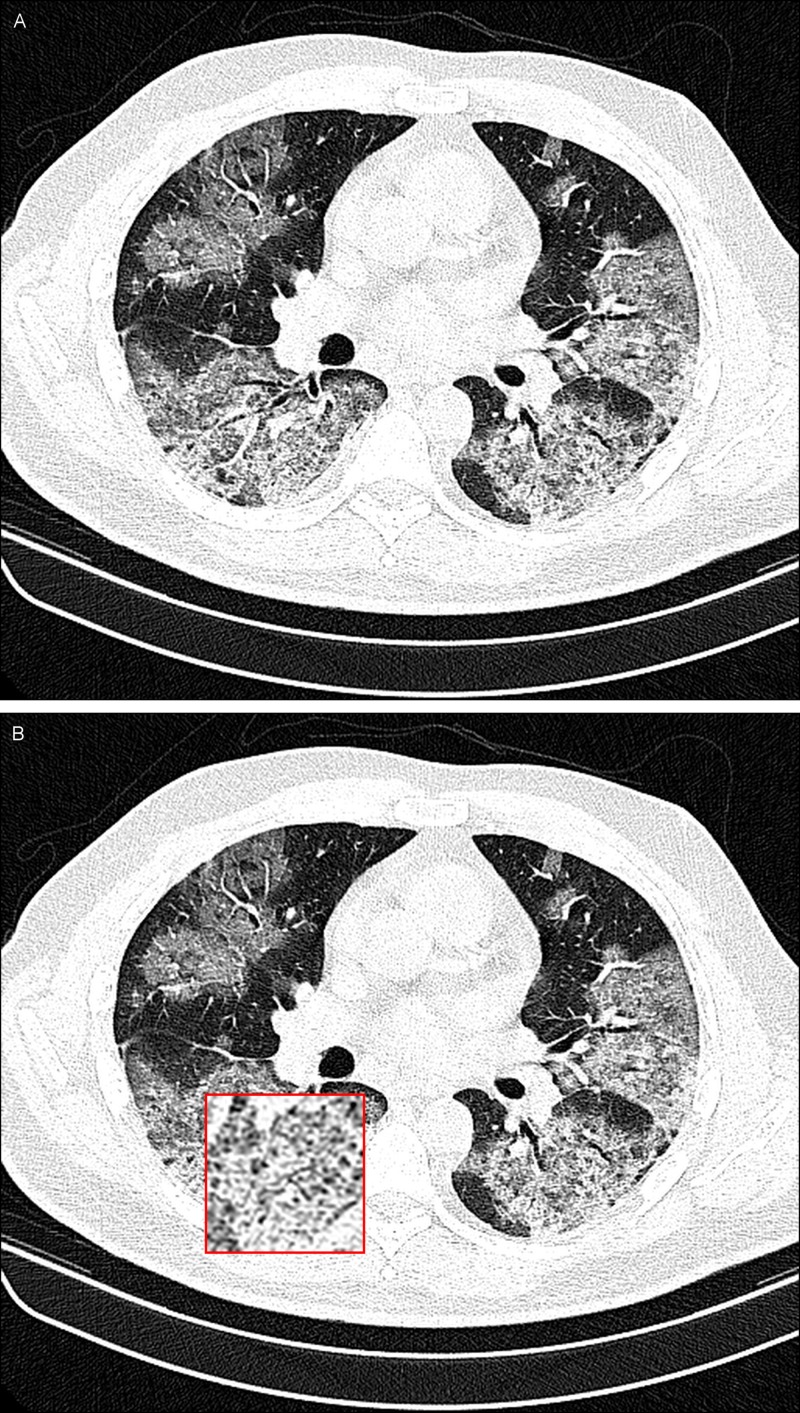
A 51-year-old man with COVID-19 infection. A noncontrast-enhanced CT image of the lungs demonstrating bilateral crazy paving in the posterior lobe of both lungs (A) and an inset magnified view of the lesion for better delineation at the location of crazy paving in the right lower lobe of the lung (B).

A 51-year-old man presented in our clinic with fever (38.7°C), cough, dyspnea, and intermittent chest pain. In his physical examination, he exhibited coarse breath sounds during auscultation. Axial thin-section unenhanced CT images obtained on March 4, 2020 show crazy paving mostly in the left and right lower lobes of the lung (Figure 2).

Case 3

Consolidation

Consolidation is detected when underlying vessels and airways are obscured due to complete replacement of air with fluid. Consolidation appears white on CT images.

**Figure 3:**
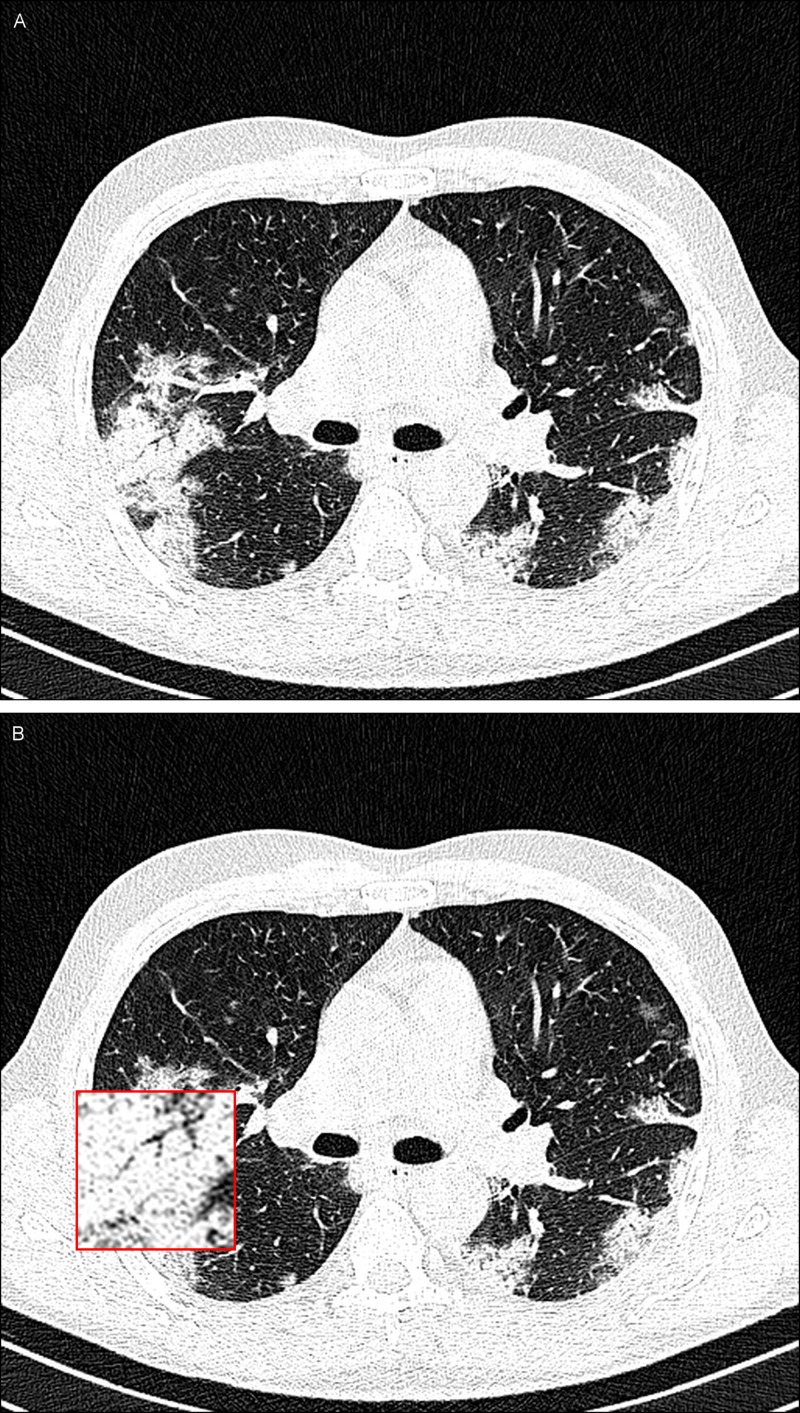
A 43-year-old man with COVID-19. A noncontrast-enhanced CT image of the lungs showing consolidation in both lungs (A) and an inset magnified view of the lesion for better delineation at the location of consolidation in the right lower lobe of the lung (B).

On March 8, 2020, a 43-year-old man from the north of Iran, one of the main epicenters of the COVID-19 infection in the country, was referred to our center after the onset of fever (38.6°C), nonproductive cough, muscle aches, and dyspnea. In his lung examination, coarse breath sounds were heard during auscultation. His transaxial unenhanced chest CT images show dense consolidation (Figure 3).

## Discussion

Given the limited number of nucleic acid testing kits, such as real-time reverse transcriptase polymerase chain reaction (rRT-PCR) assays and the probability of false-negative rRT-PCR results, chest CT imaging as a noninvasive imaging modality can be a useful tool with high accuracy for early diagnosis of suspected COVID-19 cases [7]. For this purpose it is necessary to identify common imaging patterns of these cases.

Interestingly, Fang et al. found that COVID-19 rRT-PCR sensitivity may be as low as 71% compared to the 98% sensitivity of CT for COVID-19 infection [8]. Due to the ease of access, wide availability, and rapid results of CT scanners compared to rRT-PCR kits, CT imaging is on the front line of COVID-19 screening tools. Up to the present time, most COVID-19 cases have shown pure GGO and consolidative lesions in 60% of their early chest CT imaging [5,9]. As the infection advances, CT imaging will reveal other findings such as the crazy-paving pattern.

## Conclusions

When radiologists see the common chest imaging findings of this new strain of coronavirus, they can identify COVID-19 cases in a timely manner based on the appropriate epidemiologic and demographic features. In so doing, prompt reasonable preventive action can be implemented to curtail the transmission of this enveloped positive-sense RNA virus.
